# Complete mitochondrial genome sequence of *Pleurobranchaea novaezealandiae* and *Pleurobranchaea* sp.

**DOI:** 10.1080/23802359.2016.1167636

**Published:** 2016-04-19

**Authors:** Liu Chen, Tiezhu Yang, Heding Shen

**Affiliations:** Key Laboratory of Exploration and Utilization of Aquatic Genetic Resources, Ministry of Education, Shanghai Ocean University, Shanghai, P.R. China

**Keywords:** Mitochondrial genome, *Pleurobranchaea novaezealandiae*, *Pleurobranchaea* sp., phylogenetic tree

## Abstract

The complete mitochondrial genome sequences of *Pleurobranchaea novaezealandiae* and *Pleurobranchaea* sp. are first described and analyzed in this study. It is 14,531 bp and 14,709 bp in length, respectively. The base composition of the genome with A + T bias are 66.41% and 68.36%. There are 29 noncoding regions found throughout the mitogenome of *P. novaezealandiae* and 30 noncoding in *Pleurobranchaea* sp., ranging in size from 2 to 294 bp. The phylogenetic tree based on 10 mitogenome, including 1 prosobranchia, 6 opisthobranchia and 3 pulmonata was analyzed in the paper. The results showed that the opisthobranchia and pulmonata were clustered respectively, and the *P. novaezealandiae* and *Pleurobranchaea* sp. were the closest to the *Aplysia californica* in our analysis.

*Pleurobranchaea novaezealandiae* (voucher number: ASTM-Mo-P541) belongs to Mollusc, Gastropoda, Opisthobranchia, Pleurobranchidae, which has a widespread domestic distribution, such as Bohai Sea, Yellow Sea, Paracel Islands and HongKong, and Japan is its main foreign inhabitancy. *Pleurobranchaea novaezealandiae* is characterized by small body. It presents oblong shape, with a bulge on its back and irregular mastoid processes on its surface. The front of its head extends bilaterally to form flat shape, with small serration at its edge and two pairs of antennae. It has large ovate-oblong feet, which is exposed outside the margin of mantle. It has a blue-yellow back, while its ventral side appears dark maroon.

*Pleurobranchaea* sp. (voucher number: ASTM-Mo-P547) belongs to Mollusc, Gastropoda, Opisthobranchia, Pleurobranchidae, a small creature inhabiting at shallow. It has oval shape, sharp tail and resembles slug. Besides, with a bulge on its back, its surface emerges white mastoid processes. *Pleurobranchaea* sp. is bilaterally symmetric taking pleopod as axle wire, and gills located at the front of head can assist to breathe, together with the secondary gills beneath mantle and the bilateral gills of pleopod.

The complete mitochondrial genome of *P. novaezealandiae* and *Pleurobranchaea* sp. were sequenced and characterized in this article, and the samples of *P. novaezealandiae* and *Pleurobranchaea* sp. were collected from Ganyu, Jiangshu Province, China. Before further processing, specimens were stored in ultra-low temperature freezer.

The complete mitochondrial genome of *P. novaezealandiae* and *Pleurobranchaea* sp. were 14,531 bp and 14,709 bp in length, respectively, and have been deposited in GenBank with accession No. KU365727 and No. KU365728. They consist of 13 protein-coding genes, 22 tRNA genes, 2 rRNA genes as shown in Tables 1 and 2. The ATG, ATT and TTG are used as the start codons, which are very common in invertebrates (Grande et al. 2008), but only ATP8 starts with TTG. Except for COX3 with an incomplete stop codon “T––”, the remaining protein-coding genes stop with the TAG or TAA. Using the tRNA scan-SE 1.21 (Lowe & Eddy 1997), 22 tRNA genes were found to fold into a typical cloverleaf secondary structure. The overall basic composition of the heavy strand in *P. novaezealandiae* is 36.53% A, 29.88%T, 19.67% G and 13.92% C, with an AT content of 66.41%. Similarly, the overall base composition of the heavy strand in *Pleurobranchaea* sp. is 40.02% A, 28.34%T, 18.46% G and 13.18% C, with an AT content of 68.36%. The AT content is higher than the content of GC, as generally shown in bivalvia mitochondrial genomes (Wang et al. 2010).

**Table 1.** Mitochondrial genome of the *Pleurobranchaea novaezealandiae.*

Locus	Position		Size	Codon		Anti-codon	Intergenic nucleotides*	Strand+
	From	To		Start	Stop			
tRNA^His^	19	85	67			GTG	2	L
tRNA^Gly^	88	154	67			TCC	27	L
*COX2*	182	839	658	ATG	TAA		−1	L
tRNA^Phe^	839	904	66			GAA	7	L
tRNA^Asp^	912	978	67			GTC	83	L
*Cytb*	1062	2154	1093	ATT	TAG		6	L
*ND41*	2161	2419	259	ATG	TAA		20	L
tRNA^Trp^	2440	2507	68			TCA	3	L
tRNA^Tyr^	2511	2575	65			GTA	29	L
*ND1*	2605	3493	889	ATT	TAA		117	L
*ND5*	3611	5093	1483	ATG	TAG		98	L
*ND6*	5192	5600	409	ATT	TAA		14	L
tRNA^Pro^	5615	5682	68			TGG	3	L
tRNA^Ala^	5686	5754	69			TGC	−1	L
tRNA^Leu^	5754	5813	70			ATT	22	L
l-rRNA	5836	6791	956				128	L
tRNA^Val^	6920	6985	66			TAC	43	L
*COX1*	7029	8538	1510	ATT	TAA		12	L
tRNA^Lys^	8551	8624	74			TTT	102	L
*NAD2*_b	8727	9306	580	TTG	TAG		61	L
*NAD2*_a	9368	9560	193	TTG	TAG		23	L
tRNA^Ile^	9584	9651	68			GAT	52	L
*COX3*	9704	10,478	775	ATG	T––		0	H
tRNA^Thr^	10,479	10,547	69			TGT	85	H
*ND4*	10,633	11,836	1204	ATT	TAA		59	L
tRNA^Ser^	11,896	11,956	61			TGA	24	L
tRNA^Ser^	11,981	12,033	53			GCT	97	H
*ND3*	12,131	12,458	328	ATT	TAG		30	H
tRNA^Met^	12,489	12,556	68			CAT	−2	H
s-rRNA	12,555	13,295	741				−9	H
tRNA^Glu^	13,287	13,350	64			TTC	−1	H
tRNA^Arg^	13,350	13,413	64			TCG	89	H
*ATP6*	13,503	14,067	565	ATG	TAA		13	H
tRNA^Asn^	14,081	14,146	66			GTT	0	H
*ATP8*	14,147	14,285	139	TTG	TAA		20	H
tRNA^Leu^	14,306	14,366	61			TAA	−1	H
tRNA^Gln^	14,366	14,426	61			TTG	50	H
tRNA^Cys^	14,477	14,531	55			GCA	0	L

* Positive numbers indicate the number of nucleotides found in intergenic spacers between different genes. Negative numbers indicate overlapping nucleotides between adjacent genes. +H and L indicate genes transcribed on the heavy and light strands, respectively.

**Table 2:** Mitochondrial genome of the *Pleurobranchaea* sp.

Locus	Position		Size	Codon		Anti-codon	Intergenic nucleotides*	Strand+
	From	To		Start	Stop			
*COX1*	13	133	121	ATT	TAA		164	L
tRNA^Lys^	298	363	66			TTT	95	L
*ND2*	459	1269	811	TTG	TAG		34	L
tRNA^Ile^	1304	1371	68			GAT	52	L
*COX3*	1424	2198	775	ATG	T––		0	H
tRNA^Thr^	2199	2265	67			TGT	40	H
*ND4*	2306	3500	1195	ATT	TAA		108	L
tRNA^Ser1^	3609	3667	59			TGA	68	L
tRNA^Ser2^	3736	3803	68			GCT	42	H
*ND3*	3846	4170	325	ATT	TAG		9	H
tRNA^Met^	4180	4246	67			CAT	−6	H
s-rRNA	4241	4975	735				−9	H
tRNA^Glu^	4967	5034	68			TTC	3	H
tRNA^Arg^	5038	5100	63			TCG	202	H
*ATP6*	5303	5756	454	ATG	TAA		27	H
tRNA^Asn^	5784	5850	67			GTT	3	H
*ATP8*	5854	5994	141	TTG	TAA		13	H
tRNA^Leu^	6008	6073	66			TAA	−2	H
tRNA^Gln^	6072	6132	61			TTG	294	H
tRNA^Cys^	6427	6489	63			GCA	12	L
tRNA ^His^	6502	6564	63			GTG	20	L
tRNA^Gly^	6585	6652	68			TCC	16	L
*COX2*	6669	7329	661	ATG	TAA		−1	L
tRNA^Phe^	7329	7395	68			GAA	5	L
tRNA^Asp^	7401	7467	67			GTC	45	L
*Cytb*	7513	8599	1087	ATT	TAG		6	L
*ND41*	8606	8855	250	ATG	TAA		29	L
tRNA^Trp^	8885	8950	66			TCA	2	L
tRNA^Tyr^	8953	9006	54			GTA	63	L
*ND1*	9070	9934	865	ATT	TAA		132	L
*ND5*	10,067	11,576	1510	ATG	TAG		69	L
*ND6*	11,646	12,060	415	ATT	TAA		0	L
tRNA^Pro^	12,061	12,125	65			TGG	10	L
tRNA^Ala^	12,136	12,203	68			TGC	−1	L
tRNA^Leu1^	12,203	12,265	63			ATT	12	L
l-rRNA	12,278	13,235	58				150	L
tRNA^Val^	13,386	13,450	65			TAC	46	L
*COX1*	13,497	14,706	1210	ATT	TAA		0	L

* Positive numbers indicate the number of nucleotides found in intergenic spacers between different genes. Negative numbers indicate overlapping nucleotides between adjacent genes. +H and L indicate genes transcribed on the heavy and light strands, respectively.

The phylogenetic tree (Figure 1) was generated using the MrBayes soft analyses and Maximum Likelihood method (Tamura et al. 2011) from amino acid composition of complete mitochondrial genomes. The results showed that the opisthobranchia and pulmonata were clustered respectively, and the *P. novaezealandiae* and *Pleurobranchaea* sp. were the closest to the *Aplysia californica* in our analysis. It constituted a framework for phylogeny evolution analysis, systematic classification of other euthyneurans (pulmonates and opisthobranchs) (Liu et al. 2015). The comparative mitogenomic analysis of gastropods may provide valuable phylogenetic information.

**Figure 1.**
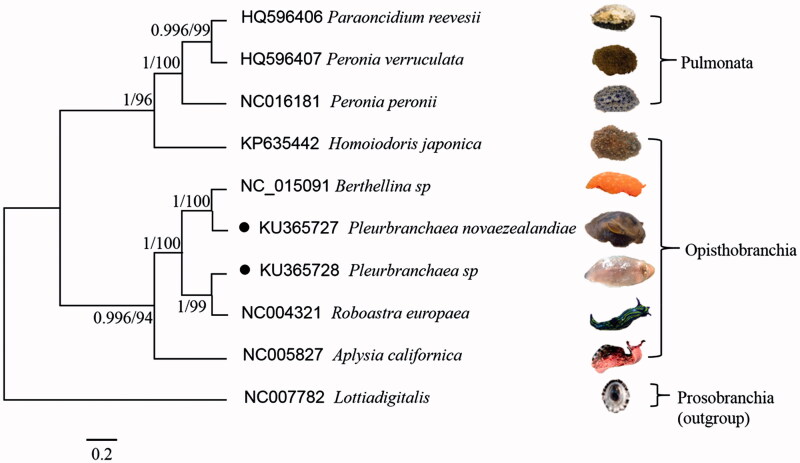
Phylogenetic tree generated using the MrBayes analyses and Maximum Likelihood method from amino acid composition of the complete mitochondrial genomes.
